# Supplementary material for: Novel co-culture plate enables growth dynamic-based assessment of contact-independent microbial interactions
Source: PLoS One. 2017 Aug 2;12(8):e0182163. doi: 10.1371/journal.pone.0182163 (PMC5540398; doi:10.1371/journal.pone.0182163)
Supplement: S1 Appendix — This document contains a parts list for the plate, a detailed explanation of the design of the plate, and a protocol for using the plate. (PDF) [file pone.0182163.s001.pdf]

S1 Appendix

Parts List

The parts list for one complete co-culture plate:

1. (8) 25 mm EMD Millipore 0.1 µm Isopore polycarbonate membranes (Fisher)
2. (1) Polypropylene Stock (1/2” thickness machined to size) (McMaster-Carr, Part No. 8782K73)
3. (48) 0-80, 9/16" Fully Threaded 18-8 Stainless Steel Socket Cap Screw (McMaster-Carr)
4. (64) No. 0, 0.062" ID, 0.156" OD 18-8 Stainless Steel Flat Washer (McMaster-Carr)
5. (12) 1-64, 3/8" 18-8 Stainless Steel Socket Head Screw (McMaster-Carr)
6. (12) 1-64, 3/16" 18-8 Stainless Steel Button Head Screw (McMaster-Carr)
7. Food-Grade High-Temperature Silicone Sheet (60A) with Acrylic Adhesive Back (custom laser cut gaskets) (McMaster-Carr, Part No. 86045K67)
8. Smooth Finish, 0.02", Transparent, Impact-Resistant Polycarbonate Sheet (cut to size with waterjet) (McMaster-Carr, Part No. 85585K17)
9. (1) Breathe-Easy ® membrane, sterile (Sigma Aldrich)

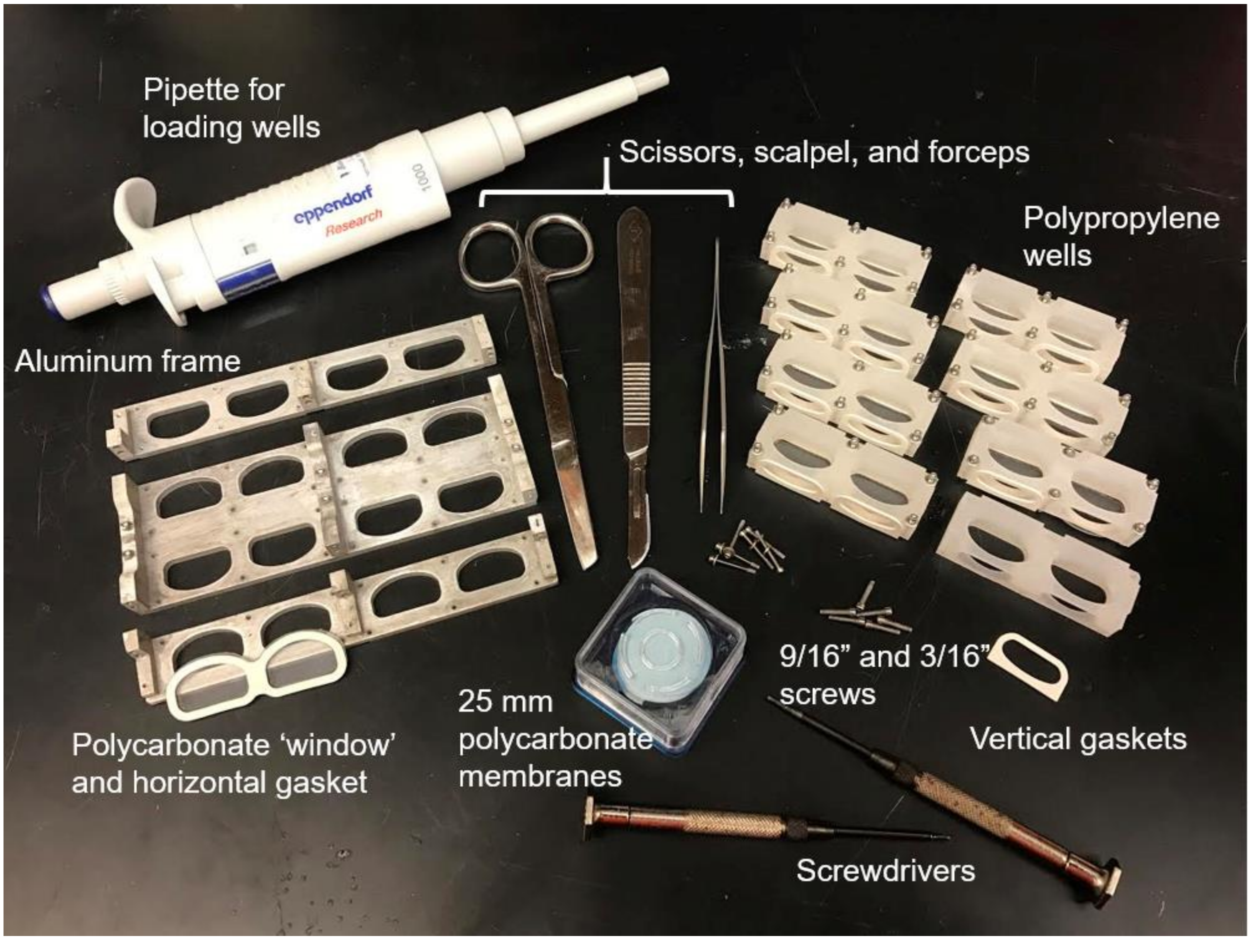

Fig. Co-culture plate parts and tools used for one complete plate.

Detailed Description of Co-culture Plate Design

Throughout this technical description, the term 'chamber' will refer to an entity consisting of two 'wells' separated by a semipermeable membrane. Thus, the fully assembled device houses 8 co-culture chambers each consisting of 2 individual wells for a total of 16 wells on the device. The disassembled device consists of 3 major parts: an 8-well central part and two 4-well side parts. Each sided part is secured to the central part by 3 stainless steel 1-64, 3/16" 18-8 stainless steel screws for a total of 6 horizontal screws (see video for visualization). The base components of the central and side parts are made of machined aluminum. Each well is made of polypropylene. All discussed parts can be autoclaved and cleaned with 70% ethanol.

During assembly, silicone gaskets are secured via acrylic adhesive around the longitudinal face of each well which uniformly clamp the polycarbonate membrane and to a clear polycarbonate well-bottoms (windows) which are placed under each well to provide a transparent bottom surface of each well for transmission based spectrophotometry. The vertically-oriented membrane is situated between 2 gaskets and secured in place when the side parts are secured to the central part. This design of the co-culture plate results in a slightly variable dimension along the short edge of the plate due to the horizontal clamping mechanism and variability in the compression of the silicone gaskets between the wells. This variation in the plate width does impact the alignment of the wells because the reading locations of the wells for a 96-well plate are within the perimeter of the co-culture plate wells.

Disassembly allows for replacement of the gaskets, when necessary, and facilitates the application of semipermeable membranes between the wells. Every part of the device is reusable with the exception of the polycarbonate membranes which need to be replaced with every use.

A video demonstrating the assembly of the co-culture plate is located at:
https://www.youtube.com/watch?v=ic3vFLgMHLo

Detailed Description of Co-culture Plate Machining

All of the SolidWorks parts and assembly files are provided in the Supplemental and at:
https://github.com/csbl/CoculturePlate

Future iterations of the plate continue to be under development, updates will be posted on this GitHub site.

Detailed Co-culture Plate Culture Protocol

Please find a complete protocol for sterilization, assembly, and plate loading below:

I. STERILIZATION

Before each experiment:

1. Assemble plate by applying all gaskets and bolts to hold wells and well bottoms in place.
2. Assure that the vertical bolts holding the polypropylene wells to the aluminum baseplate are loose, to ensure the polypropylene does not deform in autoclave due to applied tension. Steam autoclave the central and side parts at 121°C for 60 minutes in separate autoclave bags with the gaskets facing away from each other to eliminate the possibility of the gaskets adhering to each other during the autoclaving process (the non-adhesive sides of cleaned silicone will adhere due to strong Van der Waal forces if autoclaved while in contact with each other).

3. Tools and bolts can be cleaned with 70% ethanol.
4. With clean scissors, trim approximately 4 mm from the bottom of 8 circular 25 mm membranes and place in a sterile shallow dish of 70% ethanol for 10 minutes prior to use. The flat edge of the membrane will eventually be positioned along bottom edge of the well (See video).
    a. Warning: avoid touching the central portion of the membrane to avoid damaging the area essential for allowing diffusion.
    b. Membranes were ethanol-sterilized as opposed to autoclaved because autoclaving reduces the passive diffusion through the membrane and surface hydrophobicity. Data not shown.

II. ASSEMBLY

Under biosafety hood:

1. Allow to cool and remove parts from autoclave packaging. Using proper sterile technique, uniformly tighten vertical bolts around wells to create seal with bottom gaskets. Ensure each part is placed on a sterile surface (such as the inside surface of the autoclave bag after the plastic covering has been removed).
2. Using tweezers, remove membranes from ethanol dish and place on side gaskets such that the straight cut edge aligns with the bottom of the well.
3. Carefully align and clamp side part on with 1-64, 3/8" 18-8 stainless steel socket head cap machine screws (6 total for both sides). Repeat with the other side.
4. Notice that the membranes extend above the height of the device, these will interfere with the Breathe-Easy membrane. Using the tweezers to apply slight tension to the membranes, use a scalpel to trim the membranes to be flush with the tops of the wells. This will allow the Breathe-Easy membrane to be secured to the top face of the device with minimal discontinuities. Inspect membranes and gaskets for any deformity and adjust if necessary.
5. Let ethanol evaporate from membranes (approximately 10 minutes) and proceed to plate loading.

III. LOADING

The ideal volume for each well is 2 mL to prevent contact with the Breathe-Easy membrane and formation of bubbles. Wells can then be inoculated with cell according to standard microbiology protocols. The Breathe-Easy membrane then stuck to the top surface avoiding wrinkles. The membrane is slit using a scalpel blade for each well to avoid suctioning of the membrane and contact with the below culture. Care must be taken to ensure no cross-contamination occurs as a result of making the slits. The blade can be clean with 70% ethanol when necessary between wells. The co-culture plate can then be placed in a standard 96 well plate reader. The total setup time for an experiment take approximately 2 hours accounting for autoclave time.
