# Supplementary material for: Novel co-culture plate enables growth dynamic-based assessment of contact-independent microbial interactions
Source: PLoS One. 2017 Aug 2;12(8):e0182163. doi: 10.1371/journal.pone.0182163 (PMC5540398; doi:10.1371/journal.pone.0182163)
Supplement: S1 Fig — Note that the lower wells are all devoid of bacterial growth, while the well on the other side of the membrane is inoculated with an active culture of E. coli. Sterility of the wells is maintained by the membranes. (PDF) [file pone.0182163.s002.pdf]

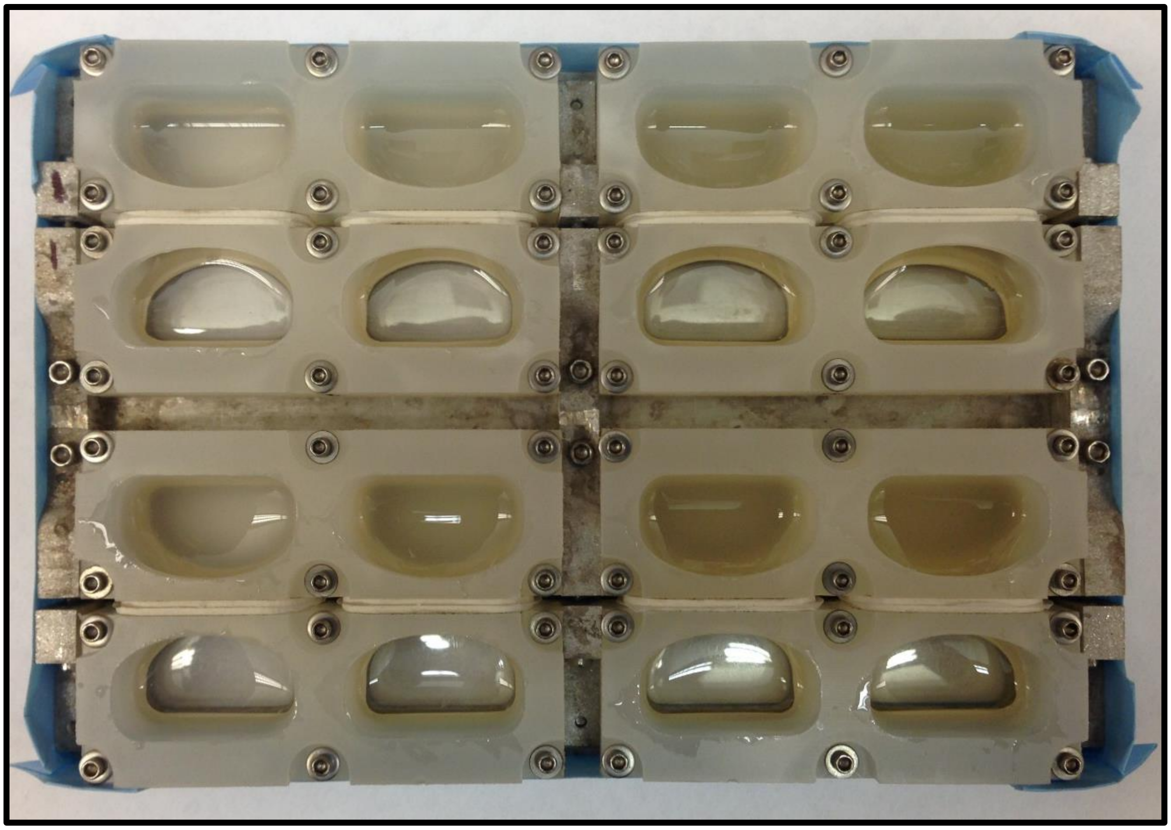

Fig S1. Endpoint image of co-culture plate after representative experiment from Fig 2. Note that the lower wells are all devoid of bacterial growth, while the well on the other side of the membrane is inoculated with an active culture of *E. coli*. Sterility of the wells is maintained by the membranes.
