# Supplementary material for: Novel co-culture plate enables growth dynamic-based assessment of contact-independent microbial interactions
Source: PLoS One. 2017 Aug 2;12(8):e0182163. doi: 10.1371/journal.pone.0182163 (PMC5540398; doi:10.1371/journal.pone.0182163)
Supplement: S2 Fig — Well 1a is PA, 1b is BC, 5a and 5b are a technical replicate of that, these are the experimental co-cultures. Wells 3a and 3b are the isolated condition of PA, 7a and 7b are the competing condition. Wells 4a and 4b are the isolated condition of BC, 8a and 8b are the competing condition. Wells 2a, 2b, 6a, and 6b are the isolated and competing conditions for PA and BC mixed, these data are not presented in the manuscript. P. aeruginosa shows clear production of pyoverdine (green pigment) in the chambers it is cultured in. The production of pyoverdine has been previously reported [41]. (PDF) [file pone.0182163.s003.pdf]

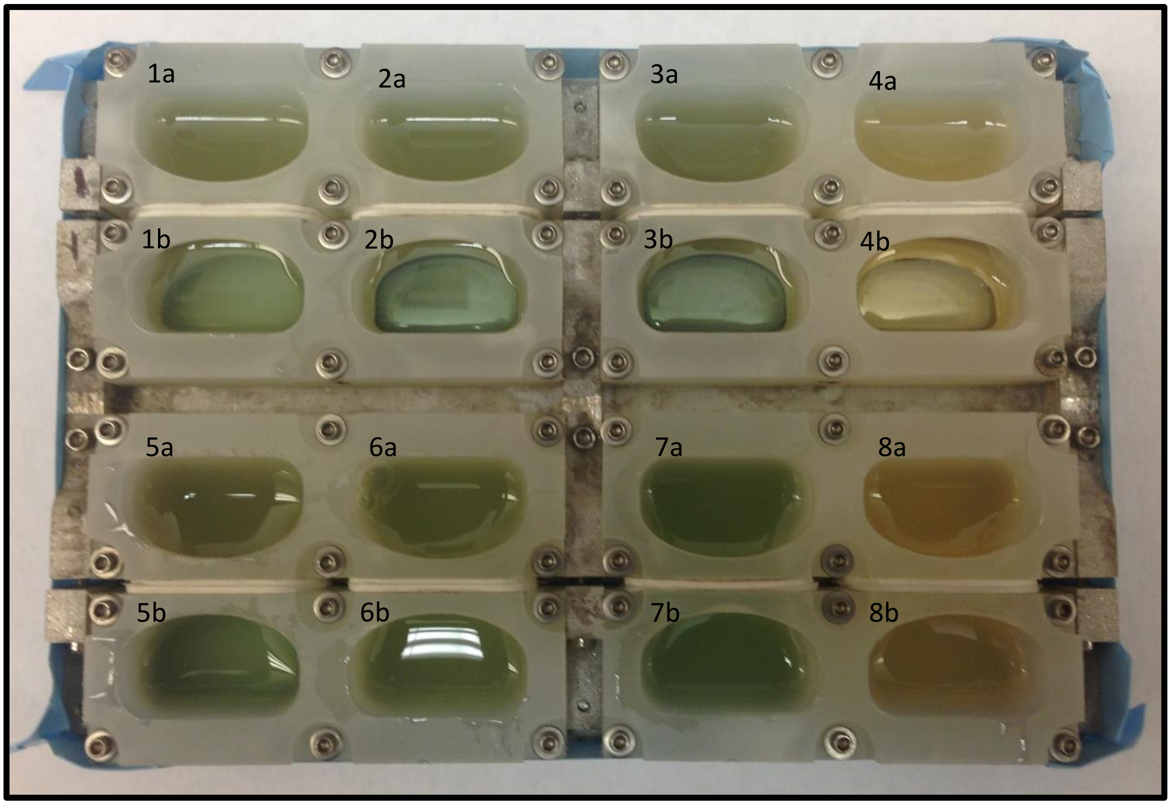

Fig S2. Co-culture of *P. aeruginosa* and B. Cenocepacia.

Well 1a is PA, 1b is BC, 5a and 5b are a technical replicate of that, these are the experimental co-cultures. Wells 3a and 3b are the isolated condition of PA, 7a and 7b are the competing condition. Wells 4a and 4b are the isolated condition of BC, 8a and 8b are the competing condition. Wells 2a, 2b, 6a, and 6b are the isolated and competing conditions for PA and BC mixed, these data are not presented in the manuscript. *P. aeruginosa* shows clear production of pyoverdine (green pigment) in the chambers it is cultured in. The production of pyoverdine has been previously reported (39).
