# Supplementary material for: Novel co-culture plate enables growth dynamic-based assessment of contact-independent microbial interactions
Source: PLoS One. 2017 Aug 2;12(8):e0182163. doi: 10.1371/journal.pone.0182163 (PMC5540398; doi:10.1371/journal.pone.0182163)
Supplement: S1 Technical Drawings — Although these drawings have the specifications for machining the parts, it is recommended that each part is machined based on the CAD files using a CNC machine. (PDF) [file pone.0182163.s004.pdf]

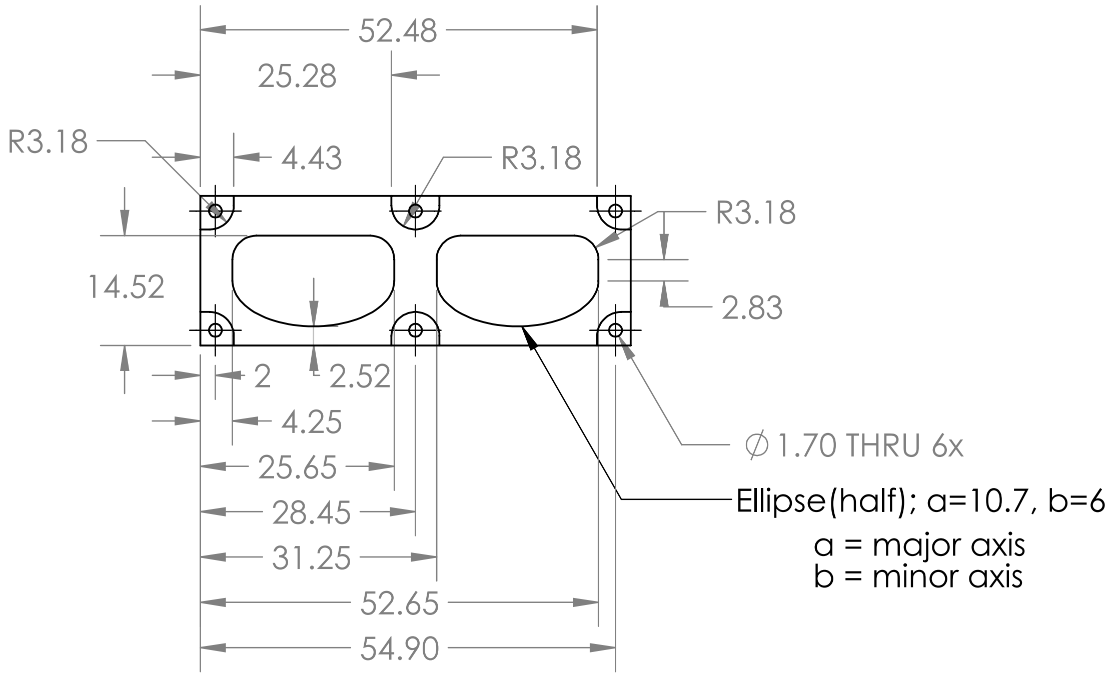

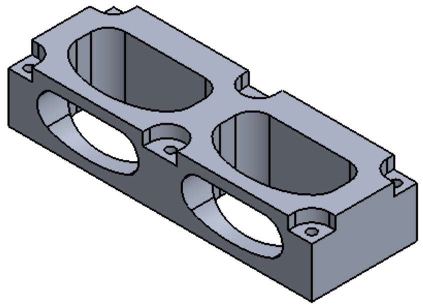

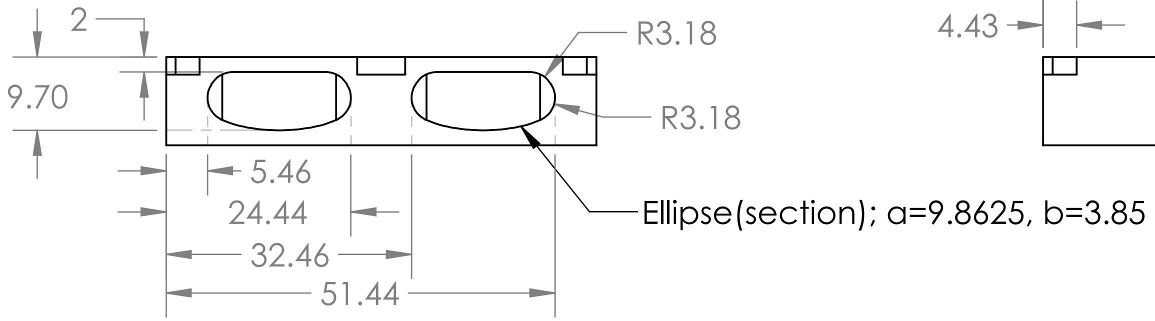

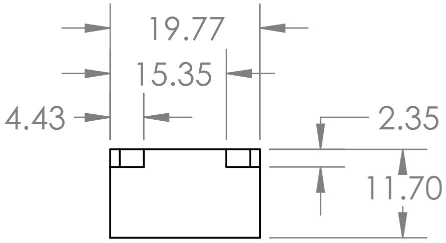

SingleChamber

DIMENSIONS ARE IN MM

Material: Polypropylene

Scale 1:1

SOLIDWORKS Student Edition.
For Academic Use Only.

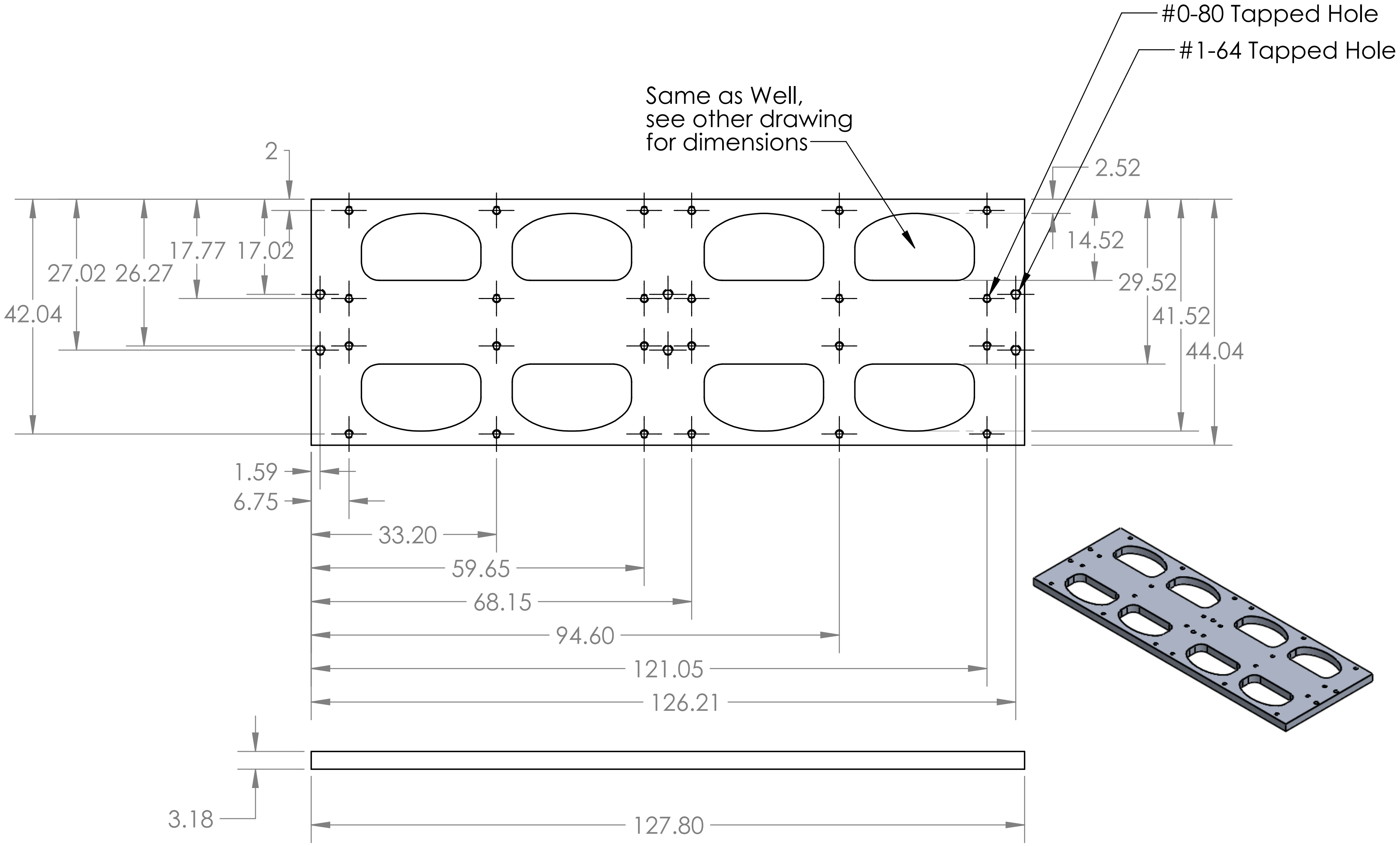

BottomPlateMiddle

Material: Aluminum
Dimensions are in MM
Scale 1:1

SOLIDWORKS Student Edition.
For Academic Use Only.

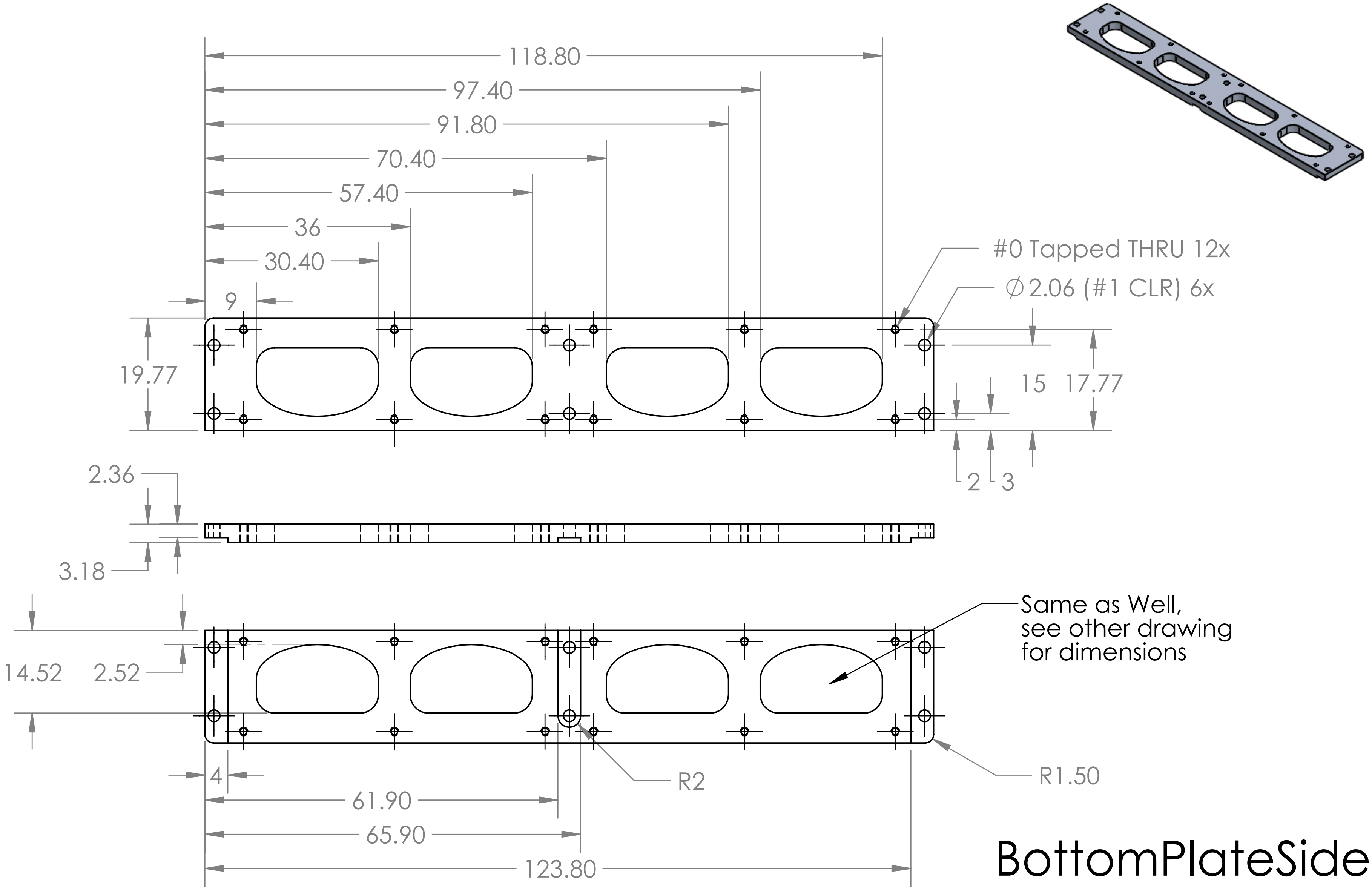

BottomPlateSide

Material: Aluminum
Dimensions are in MM
Scale 1:1

**SOLIDWORKS Student Edition.
For Academic Use Only.**

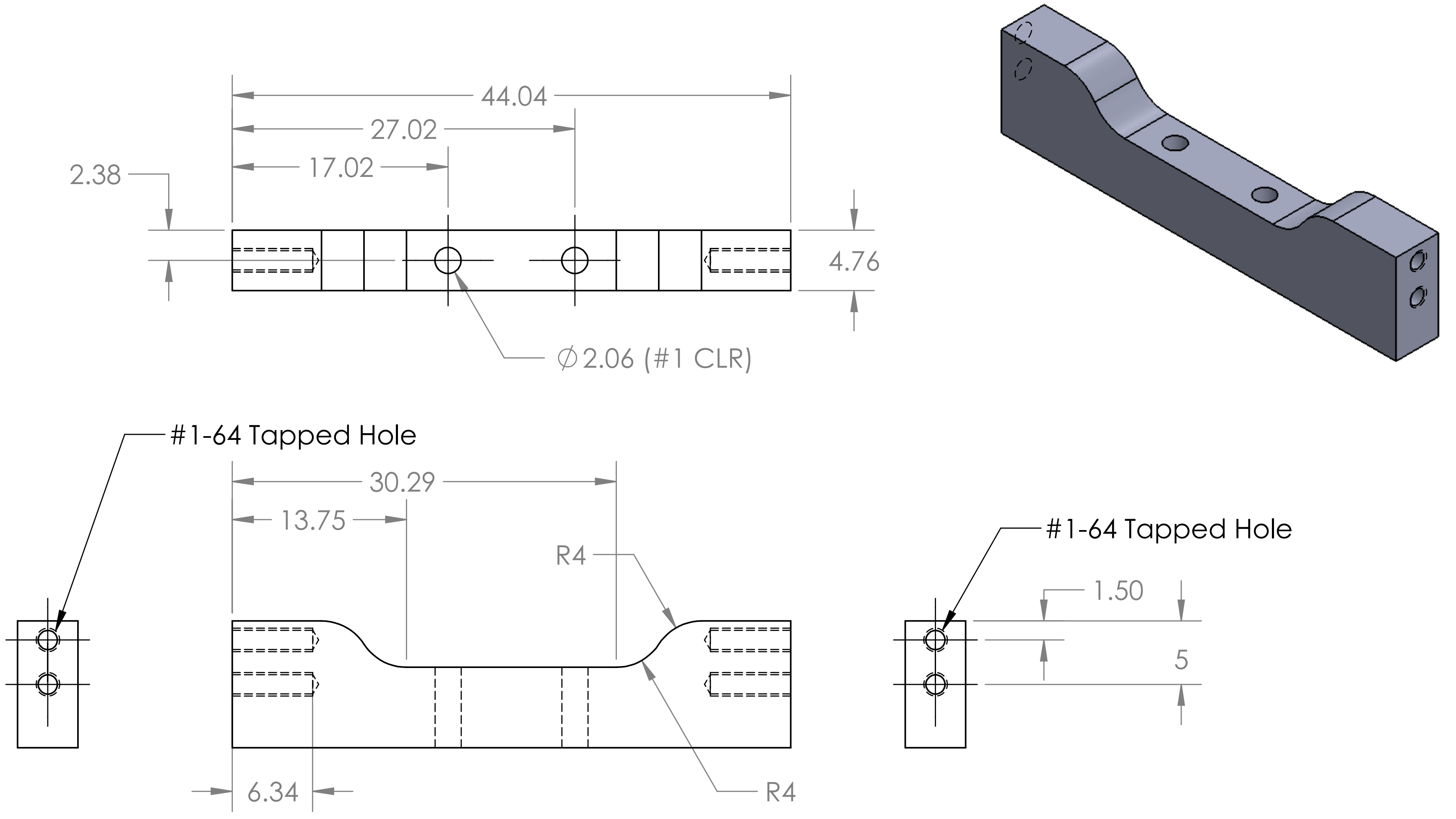

MiddlePlateSide

Material: Aluminum
Dimensions are in MM
Scale 2:1

SOLIDWORKS Student Edition.
For Academic Use Only.

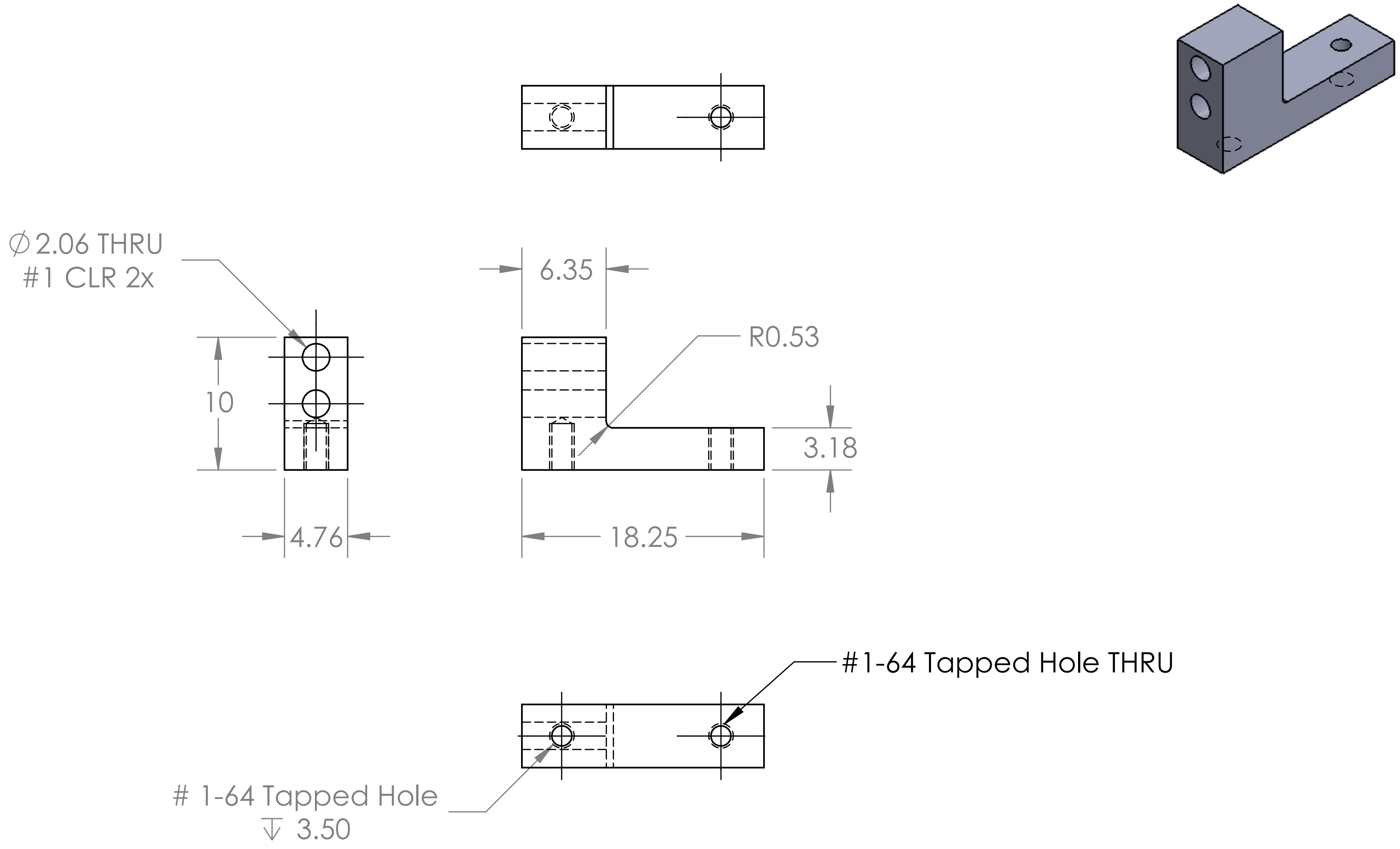

SidePlateSide

Dimensions are in MM
Material: Aluminum
Scale 2:1

**SOLIDWORKS Student Edition.
For Academic Use Only.**

2 1

B B

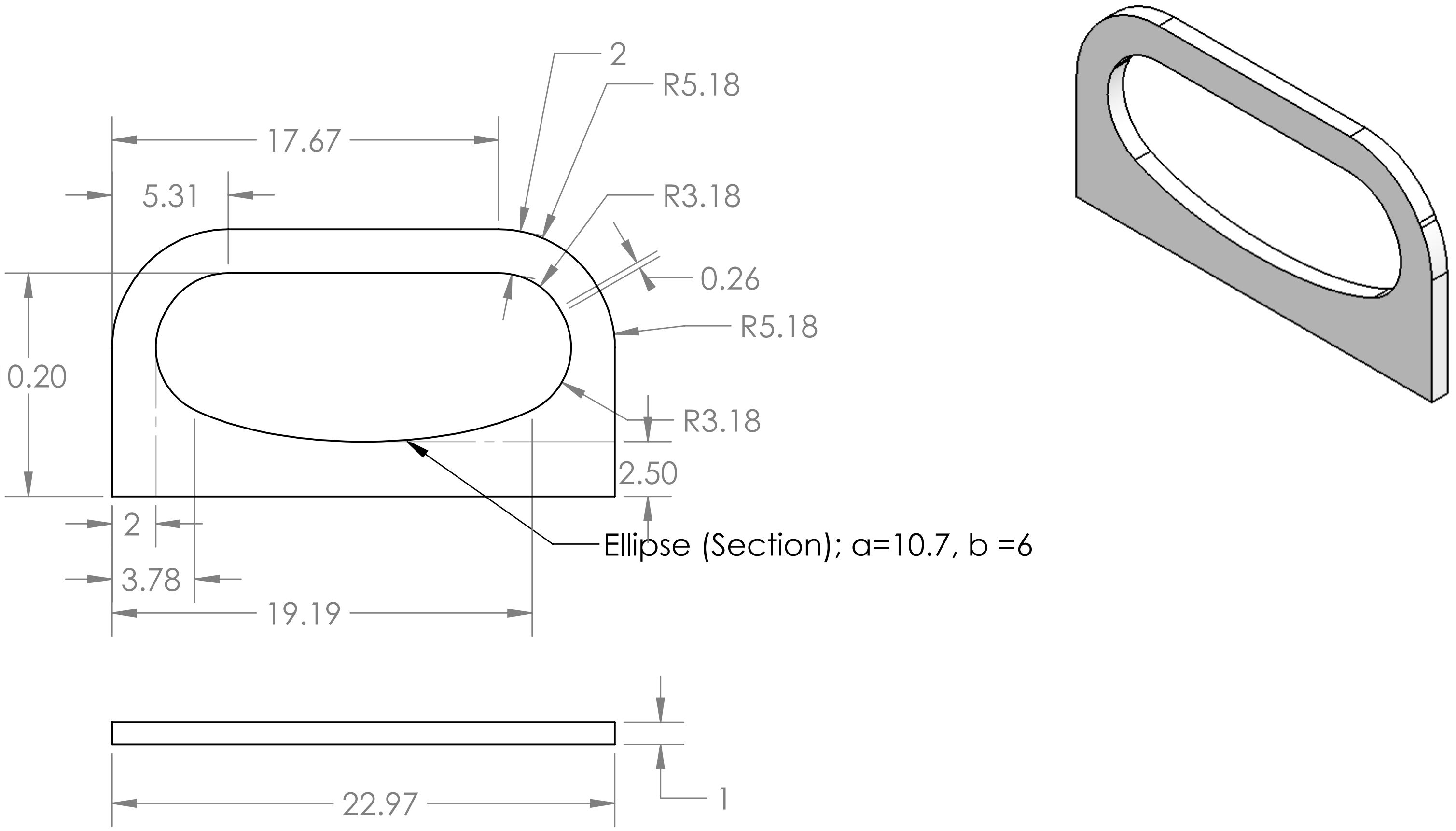

A A

Larger_membrane_gasket

Material: Silicone
Dimensions are in MM
Scale 3:1

SOLIDWORKS Student Edition.
For Academic Use Only.

2 1

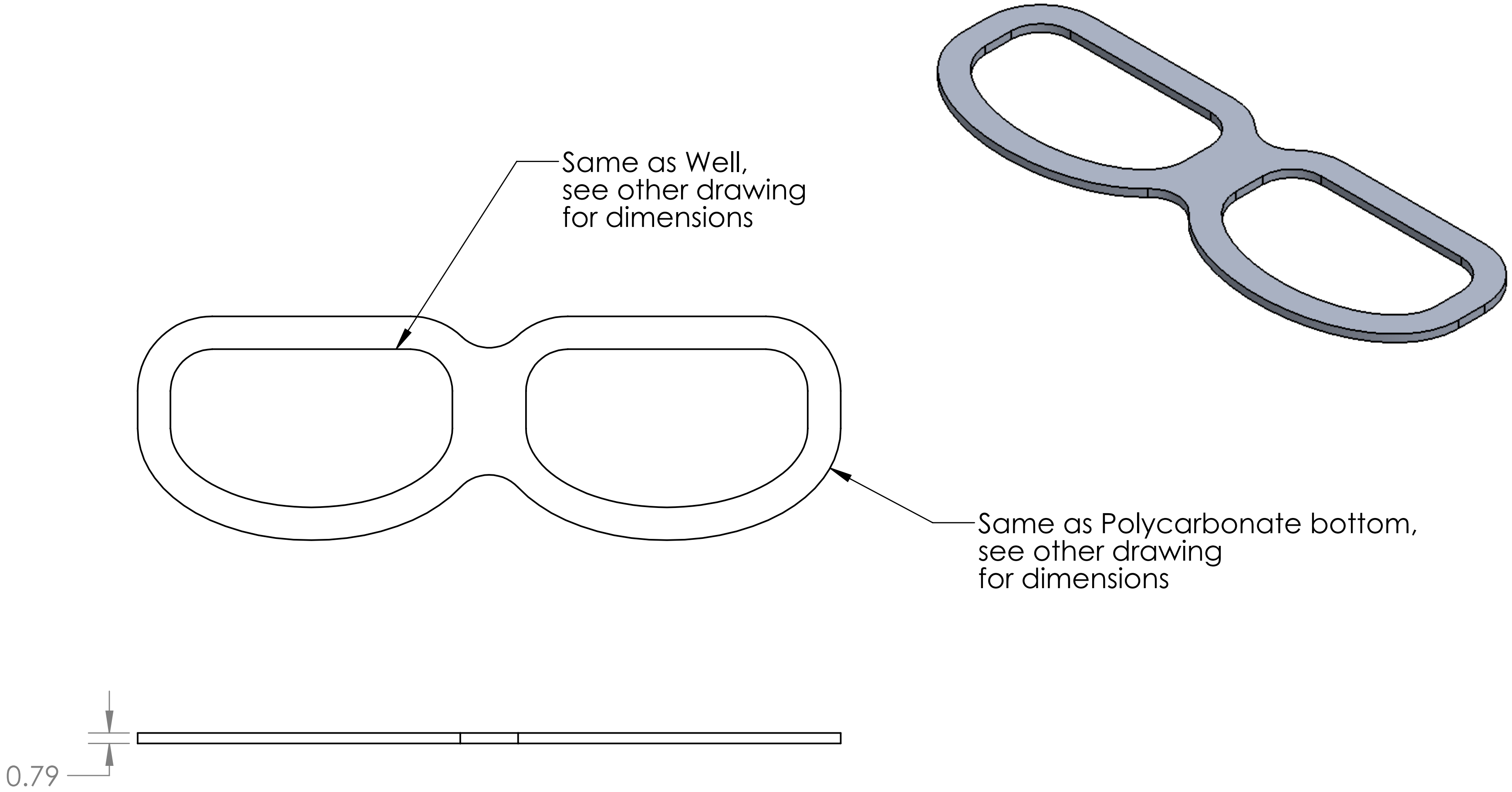

BottomGasket

Material: silicone

Dimensions are in MM

Scale 2:1

SOLIDWORKS Student Edition.
For Academic Use Only.

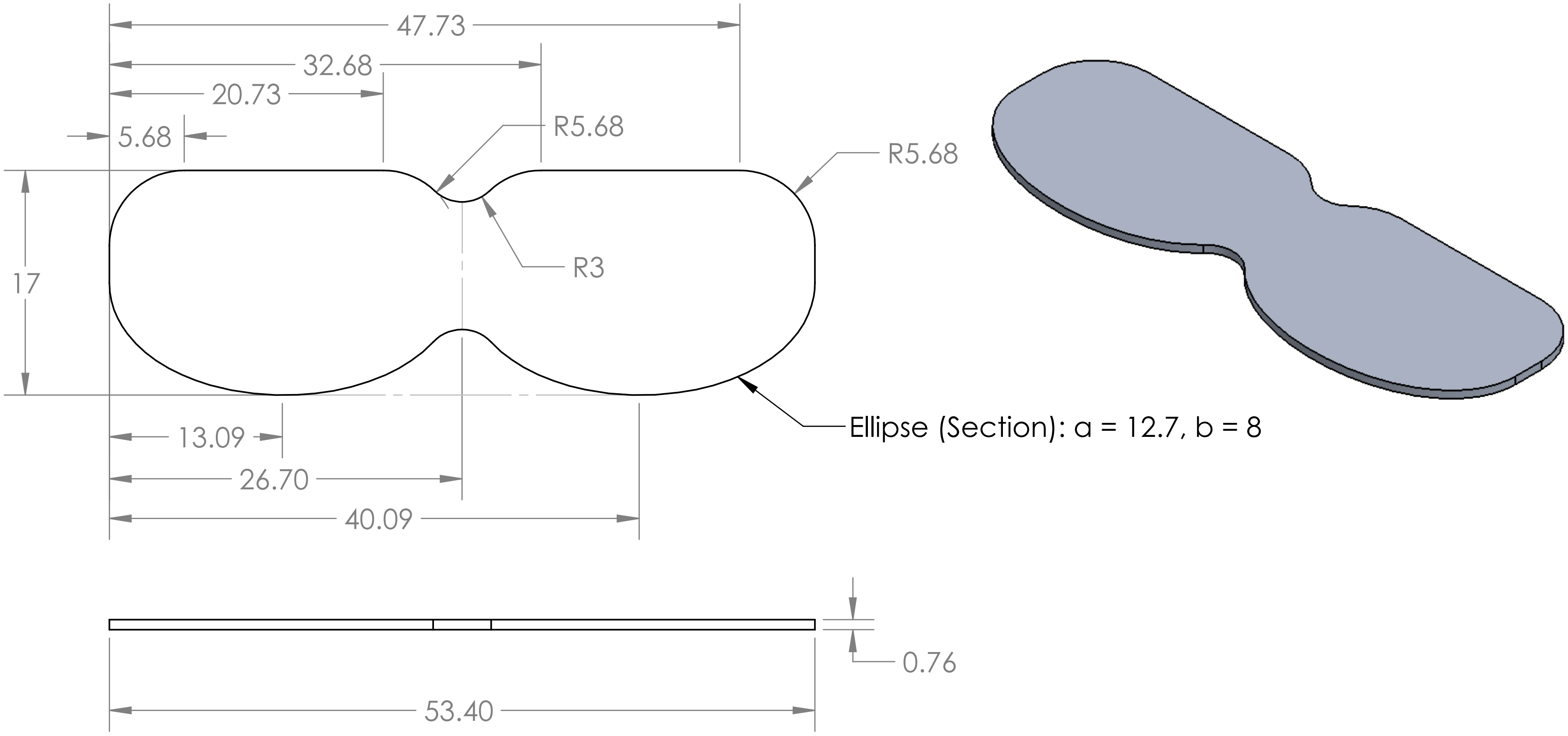

BottomPolyCarb

Material: Polycarbonate
Dimensions are in MM
Scale 2:1

SOLIDWORKS Student Edition.
For Academic Use Only.
